# A case study of the pathology on a rare case of scalp lymphangioma in an adult patient

**DOI:** 10.5339/qmj.2023.18

**Published:** 2023-08-08

**Authors:** Omar M. Shihadeh, Kazim Mohammed, Amro Elfaieg, Javeed Iqbal, Ali Raza

**Affiliations:** ^1^Neurosurgery Department, Hamad Medical Corporation, Hamad General Hospital, Doha, Qatar. E-mail: Kmohammed6@hamad.qa; ^2^Weill Cornell Medical College, Doha, Qatar; ^3^Histopathology Department, Hamad Medical Corporation, Hamad General Hospital, Doha, Qatar

**Keywords:** head and neck neoplasms/congenital, head and neck neoplasms/surgery, lymphangioma/diagnosis, lymphangioma/surgery, adult

## Abstract

Lymphangiomas are congenital malformations of the lymphatic system, mostly well-circumscribed and cystic. Although many theories were proposed to explain etiology, it is still controversial. Most of these lesions are found in the cervicofacial region, while the scalp is considered a scarce location, with only a few reported cases in the literature. Herein, we report a case of scalp lymphangioma in a 33-year-old male, which unexpectedly and significantly progressed in size over one year. The MRI scan characteristics were unique compared to the literature description of the lymphangioma, as it appeared hypointense in both T1WI and T2WI with inhomogeneous contrast enhancement, eventually consistent with lymphangioma on histopathology. The patient underwent surgical excision of the mass without any recurrence over a follow-up period of 1 year.

## Introduction

Lymphangiomas are congenital malformations that affect the lymphatic system. They are usually stable in size, well-circumscribed, cystic, and range from 1mm to 5cm. The etiology of these malformations is still a controversial topic. Lymphangioma composes around 5% of all benign lesions in children.^1^ Still, there is a wide variation in its incidence (some reported a range of 1.2 to 2.8 per 1000 people,^2^ but others reported it as low as 1 in 12,000 births).^3^ They are classified in many ways based on their histological features.^4^ They are predominantly found in the cervicofacial region. However, the scalp is considered a very rare location^5^, with less than 20 cases of scalp lymphangioma identified through an extensive PubMed search (18 of them of pediatric age). Although they are histologically benign, their location sometimes makes them behave malignantly, as they can compress critical surrounding structures.

The vast majority occur before age 2, adult-onset lymphangioma occurs exceedingly rarely, and no apparent incidence has been established.^6^ They are present mainly as a visible painless mass, but other life-threatening presentations like airway obstruction were reported.^7^ Investigations for lymphangioma may start with ultrasonography. Still, a computed tomography (CT) scan or magnetic resonance image (MRI) is advised to characterize the mass and surrounding anatomy better. On MRI, lymphangioma does not enhance with contrast and is represented by an isointense signal in the T1-weighted image and a hyperintense signal in the T2-weighted image.^8^

The definitive diagnosis relies on histopathologic features. Although many therapeutic modalities were proposed for lymphangioma, none showed superiority to the complete surgical excision. The recurrence rate depends on the treatment modality; the lesion may never recur in wide surgical excision. However, in the case of incomplete excision or other treatment modalities, the recurrence rates range from less than 10 % to 100%.^9^

The mass in our patient was proved by histopathology to be a lymphangioma, but what makes it more unique and different is not only its rare location being in the scalp but also the patient’s age (middle-aged man) as well as the MRI characteristics (hypointense in both T1WI and T2WI with inhomogeneous contrast enhancement).

## Case Presentation

**Figure 1.**
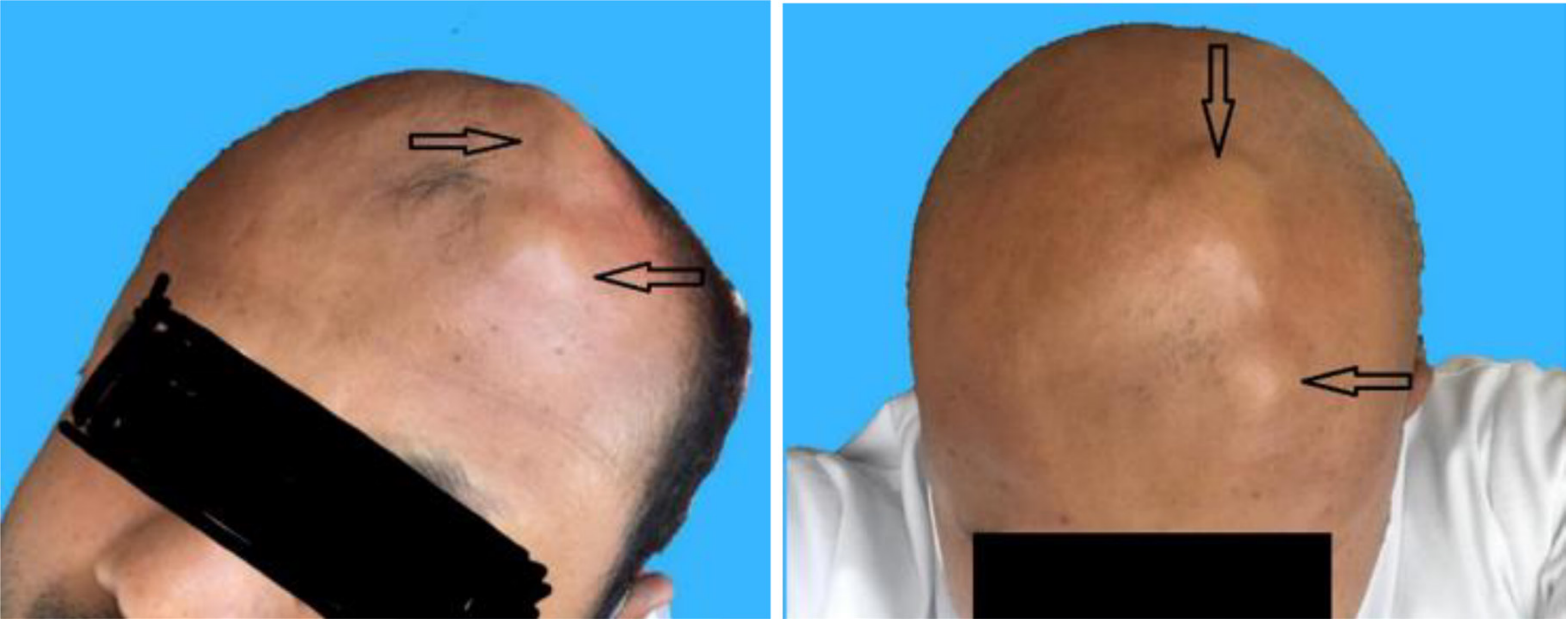
Arrows pointing to the two areas of non-tender swelling. One was anterior left frontal, about 3 cm above the eyebrow and 2 cm off midline, measuring 1*1*0.5 cm, and the second one was posterior and more medial, measuring 3*2*0.5 cm. Both lesions were multilobular and rubbery.

Our patient is a 33-year-old gentleman with no medical or surgical history who presented to the outpatient clinic complaining of scalp swelling on the forehead for one year, associated with intermittent headaches. The swelling was more evident on bending forward or lying down, while it collapsed and disappeared in a standing or sitting position. He denied any history of trauma, infection, or surgery at the site of the swelling. On physical examination, there were two areas of non-tender swelling; one was anterior left frontal, about 3 cm above the eyebrow and 2 cm off midline, measuring 1x1x0.5cm, and the second one was posterior and more medial, measuring 3x2x0.5cm. Both lesions were lobular and rubbery in consistency (Figure 1).

**Figure 2.**
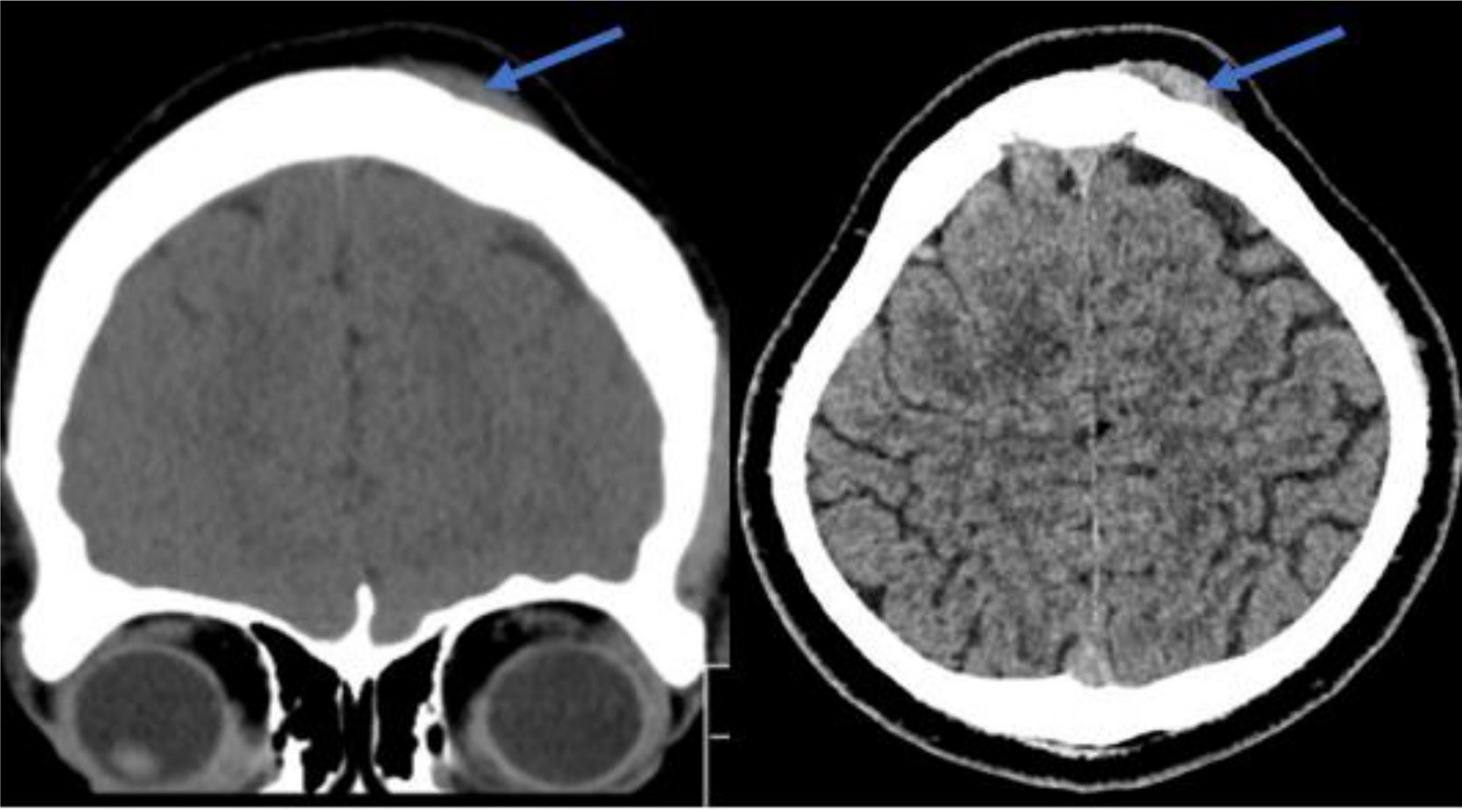
CT scan showing subcutaneous isodense lesion at the left frontal part of the scalp (blow arrows) causing erosion of the underlying bone.

**Figure 3.**
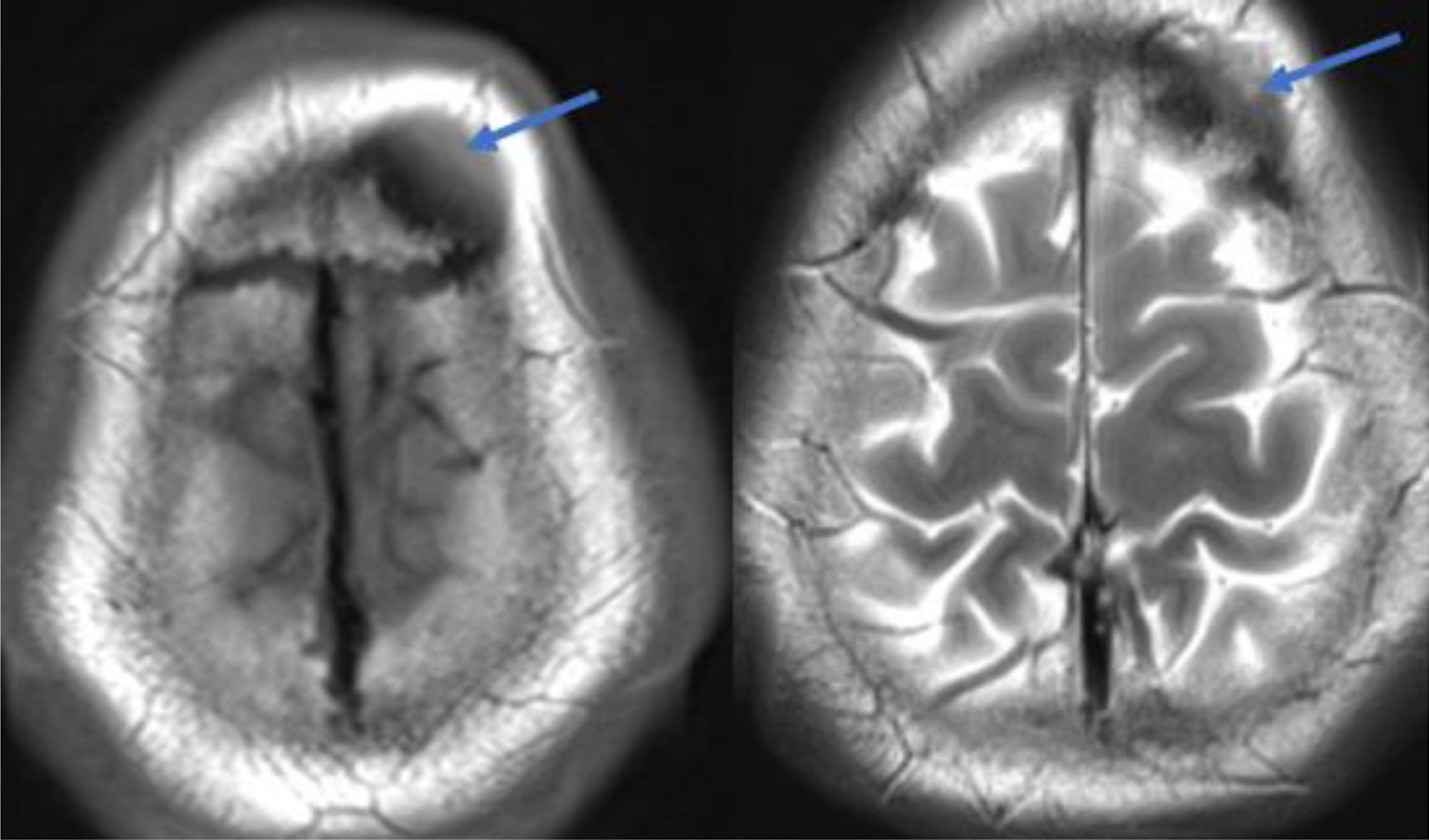
MRI T1WI (the left image) and T2WI (the right image) show the subcutaneous lesion, which is hypointense in both sequences (blow arrows).

**Figure 4.**
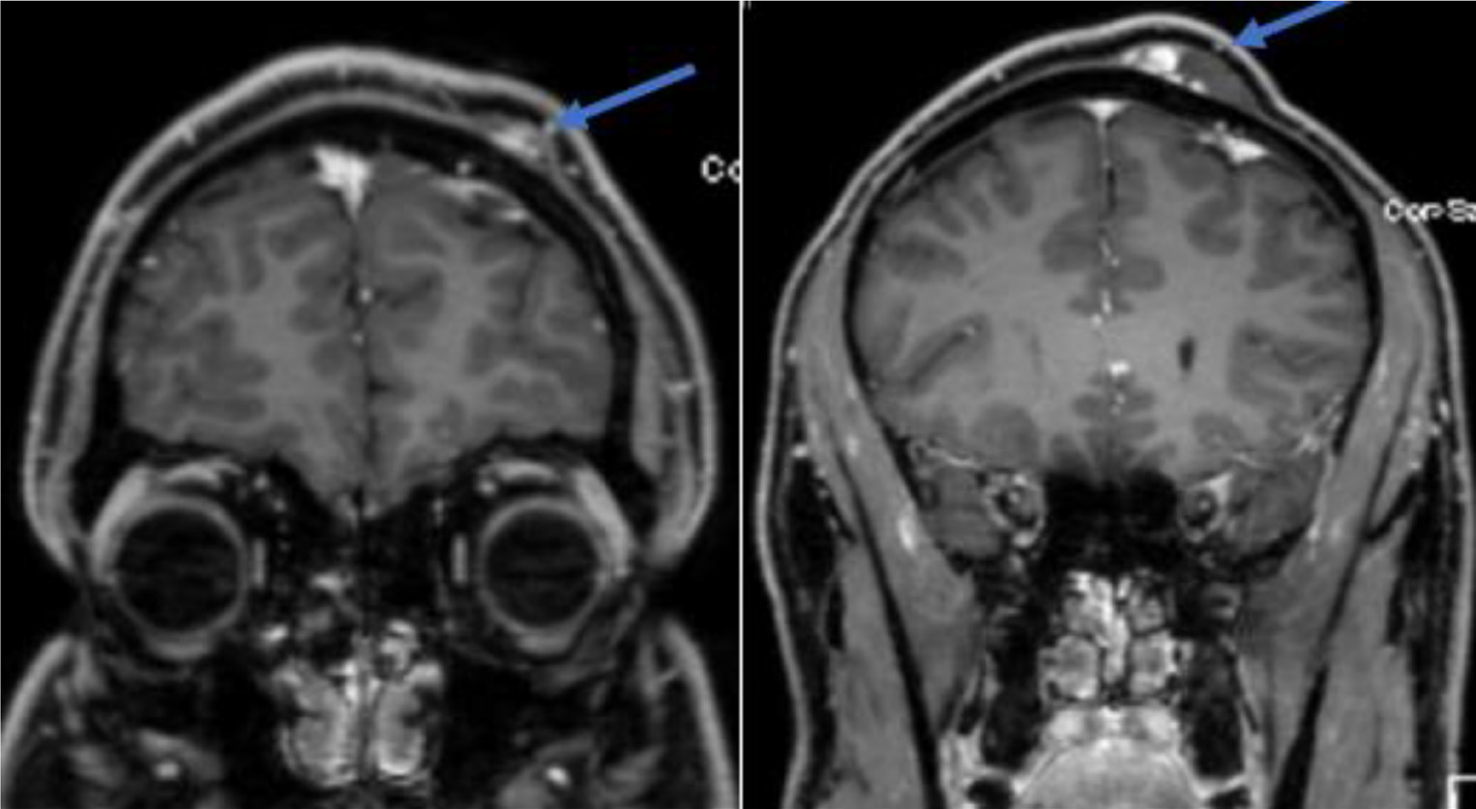
MRI T1 with contrast showing inhomogeneous contrast enhancement of the mass (blow arrows).

The neurological examination was unremarkable. Computed tomography (CT) scan of the head showed subcutaneous isodense lesions at the left frontal part of the scalp. (Figure 2). Magnetic resonance image (MRI) showed the lesions as hypointense in both T1WI and T2WI, while the contrast study revealed inhomogeneous enhancement of the lesions (Figures 3 and 4).

**Figure 5.**
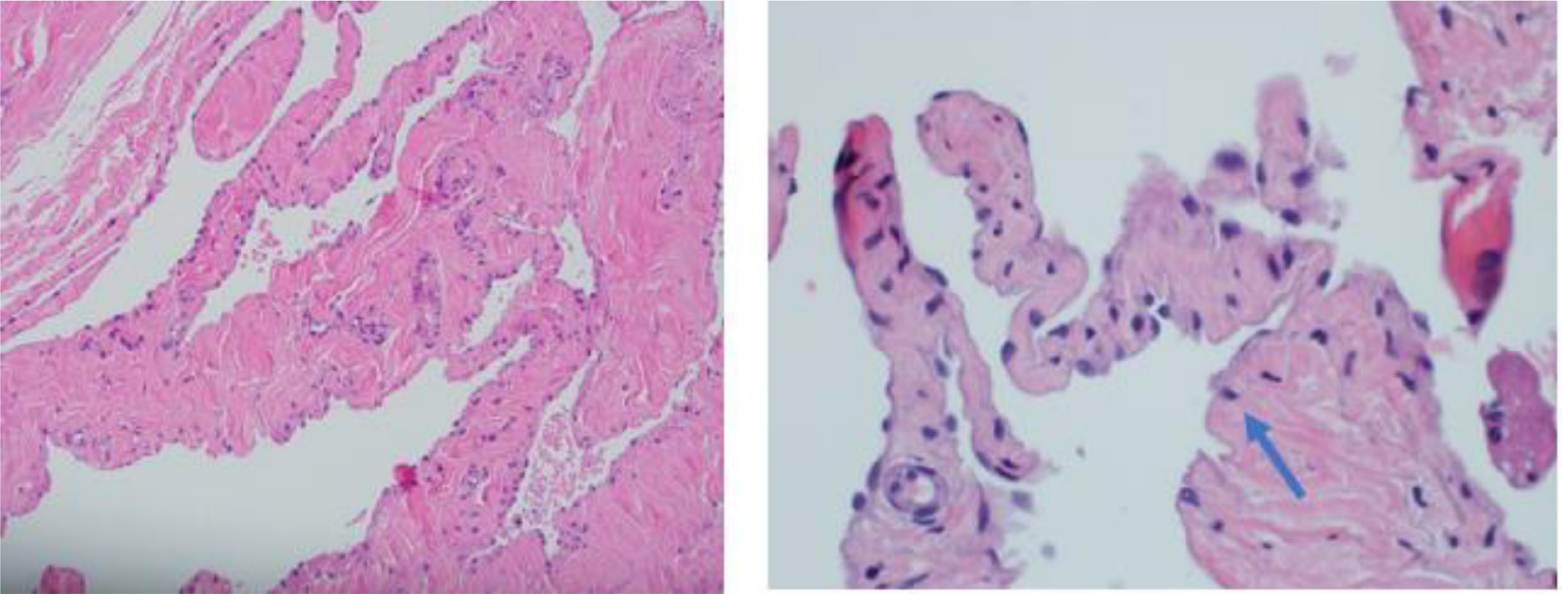
Enlarged, thin-walled, and irregular anastomosing lymphatic channels, lined by flat endothelial cells, are identified, growing in loose connective tissue. (Haematoxylin-eosin, origin, and magnification X10 (A) and X 40 (B)).

The patient underwent surgical excision of both lesions. Intraoperatively, the lesions were identified as subgaleal in location, bluish-grey in color, with no intracranial communication. They were resected totally and sent for histopathological study, which reported the masses as lymphangioma with a subclassification as cavernous type enlarged, thin-walled, and irregular anastomosing lymphatic channels, lined by flat endothelial cells are identified, growing in a loose connective tissue (Figure 5) and grossly appeared as grayish and rubbery in consistency.

The patient had a smooth post-operative recovery and was discharged in good health. On regular follow-ups in our clinics over one year, we found no evidence of any recurrence. He did not complain of headaches any further.

### Surgical Technique

1. Following skin incision up to the bone, hemostasis was achieved with Raney clips and bipolar
2. Periosteal dissection with retraction
3. First encountered larger posterior lesion subgaleal dissected and removed in one piece
4. Second encountered minor anterior subgaleal scalp lesion removed in one piece
5. No intracranial communication was identified
6. Hemostasis was achieved again
7. Barovac drain inserted, galea closed with Vicryl 2-0 suture, and the skin closed with staples

## Discussions

Lymphangiomas are mostly regarded as congenital malformations affecting the lymphatic system.^10^ They are usually stable in size but occasionally grow slowly over a long period, compressing surrounding typical structures without invasion or destruction.^11^ Lymphangiomas are often well-circumscribed, soft, and multilocular fluid-filled cystic lesions that range in size from 1mm to 5cm or more.^1,12^

Although many theories have been proposed to explain the etiology of this lymphatic malformation, it is still controversial. The first proposal states that the normal embryologic development of the primitive lymphatic channels is blocked prematurely. The second suggests discontinuity or lack of a link between the primitive lymphatic sac and the venous channels. The third theory reports that lymphatic tissue was established in the wrong area.^4^ Filston et al. reported the incidence of lymphangiomas in the range of 1.2 to 2.8 per 1000 people,^2^ but Stringel reported it to be as low as 1 in 12,000 births.^3^ They compose only 5.6% of all benign lesions in children.^1^

In a review of 153 cases of lymphangioma, Kato et al. found that 57% of the patients had a cervicofacial location of the lesion with no gender preference.^5^ In contrast, Kennedy’s 72 patients revealed 64% of craniocervical lymphangiomas with higher male predominance.^7^ As per our review of PubMed (1930 till present date), more than 90% of lymphangiomas occur before two years of age. Still, adult-onset lymphangioma rarely occurs, and no apparent incidence rate has been established.^6^ Furthermore, treatment with aggressive sclerotherapy or surgical excision during childhood is one reason for the low incidence of lymphangioma in adults.^13^ Other locations for lymphangiomas are very rare, like mediastinum and bone.^14,15^ The scalp is considered an extremely rare location for lymphangioma, and upon a PubMed search, only a few cases were reported; the first one was in 1992, as claimed by Inci et al.^16^ Hence, our case report is unique and rare in terms of the patient age (middle-aged man) as well as uncommon scalp location.

Many classifications have been proposed for lymphangioma. Wernher et al. first classified them in 1843 into capillary (lymphatic channels in the size of capillaries), cavernous (large lymphatic channel), and cystic (dilated thin-walled spaces full of serous fluid).^17^ In 1989, Kennedy suggested four types in his review: superficial cutaneous, cavernous, cystic hygroma, and diffuse systematic. The author added a new category in his classification, bringing well-localized, small, superficial lesions involving the epithelial layers with small wart-type vesicles under the superficial cutaneous lymphangiomas subtype. He suggested observation and follow-up of this subtype. In addition, the fourth subtype (the diffuse systematic), though a rare occurrence with a trend to involve large areas at times and may progress to cause generalized systemic problems with recurrent infection, cellulitis, bleeding, and thrombophlebitis, makes the treatment very difficult. Though Lymphangiomatous lesions generally result from abnormal development of a lymphatic system, based on observation in their patients, the author suggested that trauma or infection, or both furthered the development of a quiescent lymphatic system.^4^

Moreover, it is present mainly as a visible painless mass. In 65 cases described by Orvidas and 72 cases reported by Kennedy, 88% and 85% of the patients sought medical attention due to a noticeable mass without further complaints in both studies, respectively. These masses are usually soft, non-tender, painless, and lobular, although some other presentations (e.g., airway obstruction) have been reported, which may be lethal and requires urgent intervention.^7,18^

Investigations for lymphangioma may start with ultrasonography showing an echoic multilobulated cystic mass.^19^ A computed tomography (CT) scan or magnetic resonance image (MRI) is advised to characterize the mass better and, more importantly, to delineate the surrounding anatomy and its relation to the mass as a pre-operative assessment. On MRI, T1-weighted images show an isointense signal, and T2-weighted images show a hyperintense sign without contrast enhancement.^8^ Interestingly, our case had a different appearance on the MRI and did not go along with the literature description as they appeared hypointense in both T1WI and T2WI with inhomogeneous contrast enhancement. Fine needle aspiration has also been reported but with a low diagnostic value and risk for infection, hemorrhage, and recurrence.^20^ The definitive diagnosis relies on histopathologic features.

The mainstay therapy of lymphangioma is surgical excision, although it carries a significant risk of morbidity in some locations, mainly the neck. Other therapeutic modalities were proposed, but none showed superiority to the complete surgical excision, such as serial aspiration, incision and drainage, radiotherapy, and injecting the lesion with different sclerosing agents, steroids, alcohol, bleomycin, sulfate, tetracycline, and more recently, OK-432.^21^ Most studies reported good results with an excellent outcome when surgery is complete, in which the site of the lesion is the most critical determinate for a successful curative operation.^7^ The recurrence rate varies from less than 10% and may reach up to 100% in cases of partially resected complex lymphangiomas.^9^ Since we achieved complete resection of the lesions, no evidence of any recurrence was observed in our patient in the follow-up course of one year.

According to Kato et al., spontaneous regression is likely in patients older than two years and with macrocytic type within three months of the presentation.^5^ It might be rational to wait and observe for up to 2 years before deciding on surgery in the pediatric population and for one year in adults.^7^

## Conclusions

This case report highlights the importance of considering lymphangioma in the differential diagnosis of scalp swellings and even in the adult age group. Also, it draws our attention to the fact that traditional characteristics of lymphangioma in MRI might only be present in some cases. Complete surgical excision proved to be the gold standard treatment for this type of pathology as our patient has no recurrence of the mass. The location of this lesion, the patient’s age, and the MRI characteristics highlight this case as being very special and rare.

## Consent

An informed consent was taken from the patient.

## Ethical Consideration

This case report was compiled with the post-informed consent from the patient about relaying clinical history and management with a view to publication. All attached imaging and clinical material were deidentified to ensure patient anonymity. Moreover, formal approval was obtained from the Medical Research Center, Hamad Medical Corporation, with a reference number of MRC-04-22-592.

## Study Settings and Location

Neurosurgery Department, Surgical Specialty Center, Hamad General Hospital.
